# Fast Dissolving Electrospun Nanofibers Fabricated from Jelly Fig Polysaccharide/Pullulan for Drug Delivery Applications

**DOI:** 10.3390/polym13020241

**Published:** 2021-01-12

**Authors:** Thangavel Ponrasu, Bei-Hsin Chen, Tzung-Han Chou, Jia-Jiuan Wu, Yu-Shen Cheng

**Affiliations:** 1 Department of Chemical and Materials Engineering, National Yunlin University of Science and Technology, Douliu, Yunlin 64002, Taiwan; ponrasu@yuntech.edu.tw (T.P.); k23016955@gmail.com (B.-H.C.); chouth@yuntech.edu.tw (T.-H.C.); 2 Department of Nutrition, China Medical University, Hsueh-Shih Road No. 91, Taichung 404, Taiwan; jjwu@mail.cmu.edu.tw

**Keywords:** JFP, pullulan, electrospinning, fast dissolving nanofiber, drug delivery

## Abstract

The fast-dissolving drug delivery systems (FDDDSs) are developed as nanofibers using food-grade water-soluble hydrophilic biopolymers that can disintegrate fast in the oral cavity and deliver drugs. Jelly fig polysaccharide (JFP) and pullulan were blended to prepare fast-dissolving nanofiber by electrospinning. The continuous and uniform nanofibers were produced from the solution of 1% (*w/w*) JFP, 12% (*w/w*) pullulan, and 1 wt% Triton X-305. The SEM images confirmed that the prepared nanofibers exhibited uniform morphology with an average diameter of 144 ± 19 nm. The inclusion of JFP in pullulan was confirmed by TGA and FTIR studies. XRD analysis revealed that the increased crystallinity of JFP/pullulan nanofiber was observed due to the formation of intermolecular hydrogen bonds. The tensile strength and water vapor permeability of the JFP/pullulan nanofiber membrane were also enhanced considerably compared to pullulan nanofiber. The JFP/pullulan nanofibers loaded with hydrophobic model drugs like ampicillin and dexamethasone were rapidly dissolved in water within 60 s and release the encapsulants dispersive into the surrounding. The antibacterial activity, fast disintegration properties of the JFP/pullulan nanofiber were also confirmed by the zone of inhibition and UV spectrum studies. Hence, JFP/pullulan nanofibers could be a promising carrier to encapsulate hydrophobic drugs for fast-dissolving/disintegrating delivery applications.

## 1. Introduction

Fast-dissolving/disintegrating drug delivery systems (FDDDSs) is an emerging field involved in developing fast-dissolving nanofibrous films for oral administration in pediatric and geriatric patients [1,2]. In recent times, electrospun nanofibers have been extensively prepared and used in FDDDSs [3]. These nanofibers possess the dispersed drug in amorphous or nanocrystalline forms, which increases the solubility and improves the dissolution property of the water-insoluble drugs [1]. A variety of synthetic and natural polymers have been employed to produce fast-dissolving electrospun nanofibers for successful drug delivery applications including polyvinylpyrrolidone (PVP) [1], polyvinyl-alcohol (PVA) [4,5], sodium dodecyl sulphate (SDS) [6], polycaprolactone [7], chitosan [8,9], gelatin [10,11], pullulan [8], and cyclodextrin [11,12,13,14]. These polymeric nanofibers are mainly used to deliver poorly water-soluble bioactives and drugs in a rapid manner. For example, the cyclodextrin was used as a carrier and an inclusion complex to deliver a variety of poorly soluble drugs [13,14,15].

The main advantage of these nanofibers is that they can dissolve/disintegrate quickly to deliver the desired drug without swallowing and the need for water. The drug releases occur rapidly in the oral cavity, which aids rapid absorption and improves bioavailability [6]. Generally, fast-dissolving nanofibers can be prepared by several physical methods such as template synthesis, spinning, self-assembly, phase separation, and electrospinning [1]. Electrospinning is a technology in which high voltage is applied to a polymer solution or melt to create an electrically charged solution that is further ejected out from the Taylor cone to form nanofibers.

The electrospinning process is a versatile and straightforward technique for preparing polymeric nanofibers with the desired size (nanometers to micrometers) to load and release the drug in a sustained manner [16]. Electrospun nanofibers are widely used in various drug delivery systems, including oral drug delivery, transdermal drug delivery, targeted drug delivery, and tissue engineering applications [1,17]. Electrospinning also produces a thin ultrafine fiber with high surface area, volume ratio, controllable morphology, and high porosity, which can be utilized in various biomedical applications [18]. These nanofibers would be an effective alternative method for the fast disintegration of drugs than conventional strategy [3,10].

Recent studies demonstrated that the electrospinning technique has been widely employed in health care systems including drug delivery, diagnostics, theranostics, tissue engineering, and wound healing [19,20,21,22,23,24]. The multilayer electrospun membrane-based personalized reusable face mask has also been developed with photothermal application to disinfect the virus particles [25]. Among various drug delivery systems available, the electrospun fibers have drawn special attention due to having the following properties: a large surface area to load a high amount of drugs, high porosity and diameter to alter the release kinetics, site-specificity, high mechanical strength, biocompatibility, and biodegradation. The electrospun nanofibers can be implanted at the desired site for sustained drug delivery. The electrospun nanofibers are proficient in breaking down slowly as smaller fragments and degrade easily in the body without an extra effort. A distinctive coaxial electrospinning system can be useful for the fabrication of core/shell nanofibers to load a large number of free drugs. This system has been beneficial for the incorporation of chemotherapeutic drugs. Therefore, a variety of drug molecules including antibiotics, anticancer drugs, anti-inflammatory agents, proteins, and DNA are effortlessly delivered at targets from the nanofiber mats. The other nanomaterials, like nanoparticles, are not easily biodegradable and removable from the body. Generally, these nanomaterials are absorbed by the tissues and organs which creates harmful side-effects [26,27,28,29].

Over the past decades, there is growing interest in using natural polymers to develop biomaterials, such as edible films and biodegradable polymer scaffolds. Pullulan is a food-grade and water-soluble polysaccharide produced by *Aureobasidium pullulans* grown in starch and sugar media [30]. Pullulan is a natural linear polymer mainly composed of maltotriose units connected by α-(1,6) glycosidic bonds, and maltotriose consisting of three glucose units connected by α-(1,4) glycosidic bond. Pullulan is non-toxic, non-mutagenic, non-carcinogenic, biodegradable, and edible [31]. Besides, pullulan is a non-hygroscopic polymer with considerable inter-molecular mechanical strength, which can be processed into films, nanoparticles, and nanofibers [32]. Jelly fig *(Ficus pumila var. awkeotsang)*, also known as Aiyu locally, is a native plant species grown in Taiwan’s mountains. The polysaccharide extracted from jelly fig achenes (JFP) is a pectinous polysaccharide mainly composed of galacturonic acid with a low degree of methylation [33]. The JFP molecules can easily form a gel by crosslinking with multivalent metal ions [34].

**Scheme 1:**
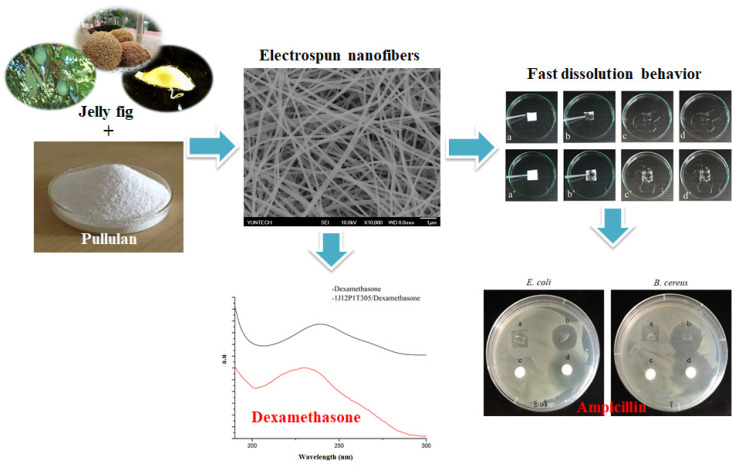
The graphical representation of electrospun nanofibers in fast-dissolving drug delivery systems (FDDDSs).

Blending different natural polysaccharides showed various synergic effects that would be useful for the preparation of nanofibers. For example, the ternary blending hydrogel made by agar/κ-carrageenan/konjac glucomannan exhibited significant improvements in mechanical and water barrier properties [35]. The blends of pectin extracted from fruit peel with sweet potato starch had a synergistic effect on the viscoelastic properties resulting in a substantial increase in the dynamic moduli of the mixture [36]. Therefore, in this study, the blending of JFP and pullulan was investigated as a copolymer system to fabricate a novel fast-dissolving nanofiber film as a drug carrier using electrospinning. Additionally, the physiochemical characterizations of developed nanofiber films were thoroughly examined and the oral treatment drugs ampicillin and dexamethasone were used as model encapsulants to determine the applicability of developed nanofibers as a carrier for FDDDS (Scheme 1).

## 2. Materials and Methods

### 2.1. Materials

The achenes of Jelly fig *(Ficus awkeotsang Makino)* were purchased from the local market and used for the polysaccharide extraction. Pullulan was procured from Tokyo Chemical Industry Co., Ltd. (Tokyo, Japan). Nonionic surfactants Triton X-100 and X-305 were bought from Sigma-Aldrich (Millipore Sigma, Merck KGaA, Darmstadt, Germany). All other chemicals used in the experiment were analytical grade also purchased from either TCI or Sigma-Aldrich if not specified.

### 2.2. Extraction of JFP from Ficus awkeotsang Makino

JFP was extracted from the *Ficus awkeotsang Makino* achenes with slight modifications [24]. Briefly, 100 mL of 0.1 M EDTA aqueous solution was freshly prepared and adjusted to pH 8 using a 4M NaOH solution. Then, 10 g of jelly fig achenes were put in 300 mL of deionized water, followed by adding 100 mL of 0.1 M EDTA solution. The mixture was then heated to 70 °C with magnetic stirring at 150 rpm for one hour. Then, the solution was filtered to remove the jelly fig achenes using a filter cloth. Then, four times the volume of ethanol (95%) was added to the filtrate to precipitate the JFP for 1 h. The JFP was obtained by filtering through a filtering screen and dried at 45 °C in an oven for 24 h to remove excess alcohol. Finally, the dried JFP polysaccharide was ground into a fine powder using a grinder and stored at room temperature.

### 2.3. Electrospinning of JFP/Pullulan Blends

A total of 1% (*w/w*) JFP powder and 6–12% (*w/w*) of the pullulan powder were dissolved in deionized water and stirred continuously for 4 h at 70 °C using a magnetic stirrer hot plate to obtain a homogeneous solution. The JFP/pullulan solution was used for electrospinning at a flow rate of 0.008 mL/min. The 10 mL plastic syringe and stainless-steel needle with an internal diameter of 0.52 mm were used to eject the solution. The voltage was applied to 27 kV. The ground wire was connected to the stainless-steel plate collector to maintain a constant electric field. The distance between the injection needle and the collection plate was 20 cm. The whole electrospinning process was carried out uniformly with a temperature of 24 °C and relative humidity of 50%. The resulting nanofibers were stored in a dry box with moisture control.

### 2.4. Morphology of JFP/Pullulan Nanofibers

The morphology of the JFP/pullulan nanofibers was observed by scanning electron microscope (SEM, JEM-2010, JEOL Ltd. Tokyo, Japan), and the photomicrographs were recorded. The nanofiber samples were adhered to the sample platform using double-sided inspection carbon tape and coated with a layer of platinum on the surface of the sample for analysis. The voltage used in this process was 15 kV. The magnification of the micrographs was ranging from 3000–10,000×. The average diameter of the nanofibers was estimated from 50 SEM images using Image J software.

### 2.5. Fourier Transform Infrared (FT-IR) Spectrometer Analysis

FT-IR spectrometer (Spectrum one, Perkin Elmer Inc., Waltham, MA, USA) was used to analyze the structure and functional groups of polymers. The analysis was performed by scanning the sample in the range of 400–4000 cm⁻^1^. The two characteristic peaks of 1615 and 1744 cm⁻^1^ were used to analyze the structure of the JFP and the peak areas were used to determine the degree of methylation (DM) of JFP.

### 2.6. Thermogravimetric Analysis (TGA)

Thermogravimetric analysis (TGA, TGA-4000, Perkin Elmer Inc., Waltham, MA, USA) was conducted to assess the thermal stability and composition of materials. 8 to 10 mg sample was kept in a ceramic tray used to measure thermal stability from room temperature to 800 °C.

### 2.7. X-ray Diffraction (XRD) Study

X-ray diffraction study (MiniFlex II, Rigaku Corp., Tokyo, Japan) was performed to analyze the phase recognition and crystallinity of the sample. The maximum employed power of the XRD spectrometer was 18 W (60 kV-300 mA). The scanning range and scanning rate were 5°–90° and 2–10°/min, respectively.

### 2.8. Tensile Strength Analysis

The texture analyzer (iDealTA, Horn Instruments Co., Ltd. Taoyuan, Taiwan) was used to examine the tensile strength of the nanofiber membrane. A test strips of nanofiber membrane (2 × 1 cm^2^) was strained at a 1 cm/min pulling speed until rupture. The obtained tensile strain and elongation ratio were used to investigate the influence of JFP addition to pullulan on mechanical properties.

### 2.9. Water Vapor Permeability (WVP) Measurement

The water vapor permeability (WVP) was measured using the method based on the standard ASTM E96-80 (ASTM, 1987) [37]. The experiment was carried out gravimetrically at 25 ± 1 °C. Briefly, the pre-weighted nanofiber samples were mounted over the cups, then anhydrous CaCl_2_ (0% RH) was placed inside the cup, and a saturated NaCl solution (75% RH) was added to the glass chamber to maintain 75% relative humidity (RH) gradient across the nanofibers. Under steady-state conditions, the permeable membrane was weighed at regular intervals for 24 h. The water vapor permeability of the samples was evaluated in triplicate using the following equation:

(1) $$\mathrm{WVP}\;=\;\frac{w\cdot L}{A\cdot t\cdot ∆p}$$

where w is the weight of the water permeated through the sample (g), L is the thickness of the sample (mm), A is the permeation area (m^2^), t is the time of permeation (h), and ∆*p* is the water vapor pressure difference between the two sides of the sample (kPa).

### 2.10. The Water Solubility or Dissolution Property of JFP/Pullulan Nanofibers

The water solubility of nanofibers was determined at room temperature (25 ± 1 °C) by adding water dropwise on pullulan and JFP/pullulan nanofiber samples (1.5 cm × 1.5 cm) placed in glass Petri dishes. The dissolution property was recorded by continuous shooting mode [8].

### 2.11. Test of In Vitro Drug Release from JFP/Pullulan Nanofibers

To test the applicability of prepared JFP/pullulan nanofiber membrane in the FDDDS, ampicillin and dexamethasone were employed as model encapsulants for in vitro release studies. The in vitro release profile of ampicillin and dexamethasone was assessed at 25 ± 1 °C. A total of 1 mg ampicillin and 40 mg dexamethasone were dissolved into a 10 mL JFP/pullulan solution before electrospinning JFP/pullulan nanofibers. The in vitro release of ampicillin and dexamethasone from the prepared nanofiber membrane was examined by antibacterial test and spectrometry. Briefly, 4 mg of the drug-loaded or blank JFP/pullulan nanofiber was dissolved in 10 mL of distilled water, and then a 1 mL solution sample was taken for the UV/Vis spectrometric analysis with scanning wavelength from 190 to 300 nm. The prepared nanofiber membranes would be a useful candidate for oral drug delivery applications particularly, in pediatric and geriatric patients. Therefore, the water was used as a release medium. Further, the antibacterial activities of the drug-loaded and blank JFP/pullulan nanofiber membranes were assessed by the disc diffusion method using Gram-negative (*E. coli*) and Gram-positive bacteria (*B. cereus*) grown on Luria broth agar plates.

## 3. Results and Discussion

### 3.1. Properties of Electrospinning Solutions

The viscosity and conductivity are the main properties to access the electrospinnability of a solution [38]. These two values are usually measured before electrospinning. The viscosity of the solution plays a vital role in electrospinning which determines the fiber diameter. A very low viscosity disrupts the polymeric filaments and a very high viscosity will not be suitable for electrospinning due to the high cohesion and flow instability of polymer solution. Generally, a viscosity of 1–20 poise and surface tension of 35–55 dyn/cm^2^ is considered suitable for the electrospinning process [39,40]. The electrospinning process primarily involves electrical charge transfer from the polymer droplet placed at the end of the injection needle. The conductivity designates the presence of electric charges on the surface of the polymer solution and regulates the propensity to form nanofibers during electrospinning. The addition of an electrolyte can improve the conductivity of the polymer solution which provides the elongation capacity of the solution to form smooth nanofibers with the desired diameter. Hence, a minimum electrical conductivity is essential for the electrospinning process [39,41].

**Figure 1:**
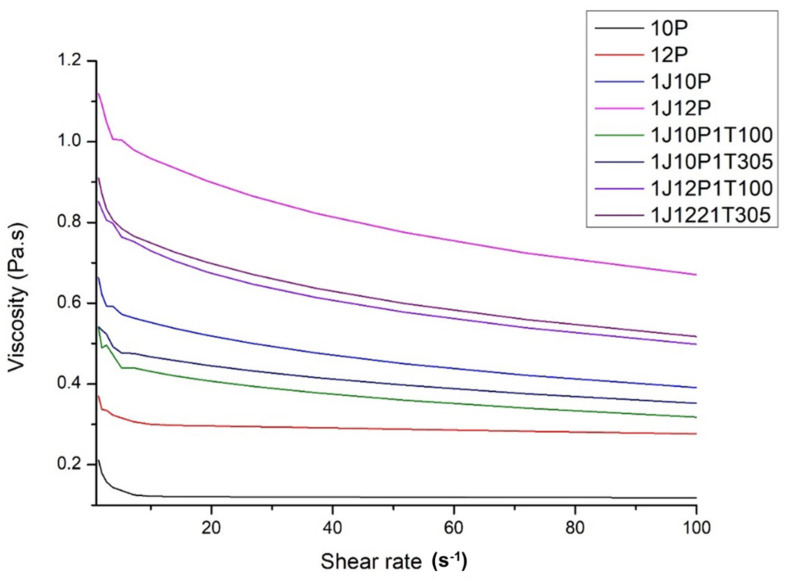
Viscosity as a function of a steady shear rate for various concentrations of jelly fig polysaccharide (JFP) and pullulan.

Besides, nonionic surfactants were commonly used to enhance the electrospinnability of an aqueous mixture of polymers [42,43,44]. Therefore, surfactants Triton X-305 and Triton X-100 were investigated for their influence on the properties and electrospinnability of JFP/pullulan solutions. The results (Figure 1 and Table S1) showed a similar trend with other literature [45]. For pure pullulan solutions, the viscosity increased with the increasing rate of shear force. However, as the shear force increases, the viscosity was decreased accordingly. The minimal change in viscosity values from 0.212 to 0.142 Pa·s was observed for the 10% (*w/w*) pullulan solution and from 0.391 to 0.257 Pa·s for the 12% (*w/w*) pullulan solution as the shear force rate decreased from 1 to 100 L/s. The 10% (*w/w*) and 12% (*w/w*) pullulan solutions exhibited a Newtonian behavior within this shear rate range. While 1% (*w/w*) JFP was added to the 10% (*w/w*) and 12% (*w/w*) pullulan solutions, the viscosity increased significantly from 0.626 to 1.162 Pa·s, and the shear thinning behavior was observed. This result indicated that adding 1% JFP to the pullulan solution increased the chain-chain interaction or entanglement between JFP and the pullulan macromolecules. The results also showed that the addition of both surfactants decreased the viscosity of the JFP/pullulan solutions, but the surfactant added solutions still exhibited shear-thinning behavior and considered as pseudoplastic fluids.

The 10% pullulan solution showed the lowest conductivity of about 8.8 μS/cm. However, the conductivity increased to 27.5 μS/cm, while the concentration of pullulan increased to 12%. Then, the addition of 1% (*w/w*) of JFP to 10% and 12% pullulan solutions showed an increased conductivity from 302.8 and 407.3 μS/cm. The data revealed that JFP has polyelectrolyte properties. When the JFP/pullulan solutions were added with Triton X-305 and Triton X-100, the conductivity was increased because of the higher hydrophilic-lipophilic balance (HLB) value of the added surfactants [46]. The HLB values of Triton X-100 and Triton X 305 were 13.4 and 17.3, respectively. At higher HLB values, the conductivity of the solution gets increased due to the association of the hydrophilic head with the water molecule [45]. The HLB is a quantity of the hydrophilic portion of a surfactant molecule that delivers a simple procedure to designate the strength of the hydrophilic-lipophilic balance of surfactant [47,48]. The balance of the hydrophilic and lipophilic groups of the surfactant molecule is similarly imperative viscosity and conductivity of the electrospinning solution [47]. The addition of nonionic surfactants is crucial due to the presence of a hydrophilic head to increase the conductivity of the solution [45]. Therefore, a negatively charged polyelectrolyte (the JFP) polymer was added to the solution to increasing the conductivity of the electrospinning solution.

### 3.2. Morphology of Nanofibers

**Figure 2:**
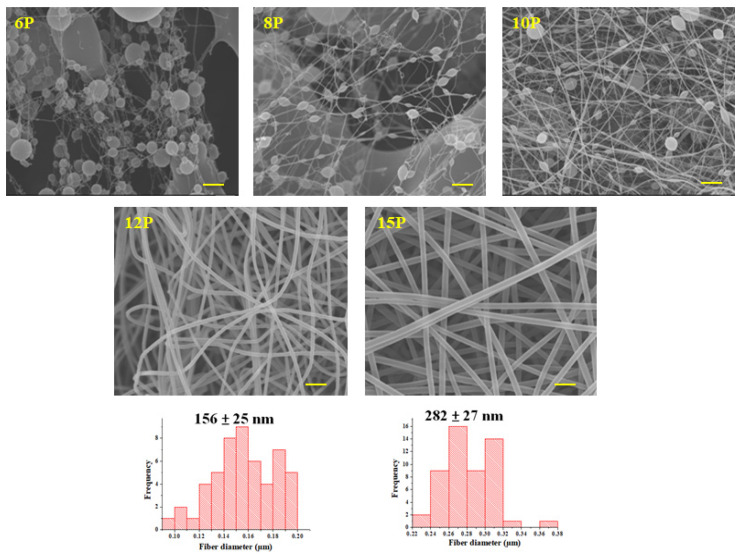
SEM images of pullulan nanofibers (6–15% *w/w*) showing the morphology and diameter distributions: 6P: 6% pullulan; 8P: 8% pullulan; 10P: 10% pullulan; 12P: 12% pullulan and 15P: 15% pullulan. Scale bar 1 µm; 10,000×.

**Figure 3:**
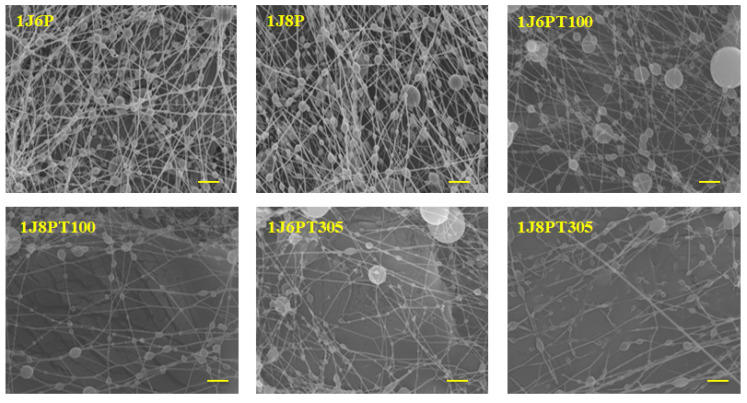
SEM micrographs showing the morphology and diameter distributions of 1% JFP/pullulan (6–12%) nanofibers along with surfactants (Triton X-100 and Triton X-305). Scale bar 1 µm; 10,000×.

**Figure 4:**
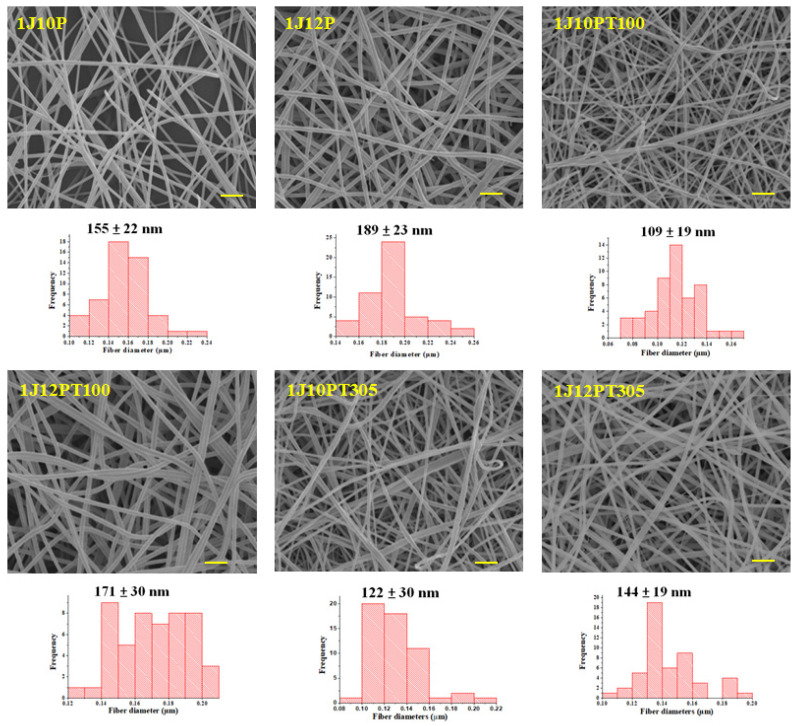
SEM micrographs showing the morphology and diameter distributions of 1% JFP/pullulan (10–12%) nanofibers along with surfactants (Triton X-100 and Triton X-305). Scale bar 1 μm; 10,000×.

The morphology and diameter distributions of pullulan nanofibers are depicted in Figure 2. The pure pullulan solution of 6%, 8%, and 10% (*w/w*) produced beaded fibers due to the lack of polymer chain entanglement at those concentrations [49]. The increased concentration of pure pullulan by 12% to 15% produced ultrafine fibers with diameter distributions of 156 ± 25 to 282 ± 27 nm. The results revealed that the polymer concentration is crucial to obtain continuous uniform nanofibers. The JFP 1% (*w/w*) was blended with 10% and 12% (*w/w*) of pullulan to obtain ultrafine fibers without beads (Figure 3). The addition of JFP 1% (*w/w*) to 10% and 12% pullulan solution increased the fiber diameters from 155 ± 22 nm to 189 ± 23 nm (Figure 4 top left and middle). These observations indicated that the addition of JFP to the pullulan solution enhanced the chain entanglement and interaction between the two polymers. The addition of JFP increased the conductivity of the spinning dope and enhanced the movement of electrostatic charges on the surface of the polymer jet. The SEM images also showed that the diameters of nanofibers produced from solutions with surfactants were lower than those without surfactants (Figure 4). For example, the diameters of nanofibers made from the solution of 1% JFP and 12% pullulan were decreased from 189 ± 23 nm to 144 ± 19 nm when Triton X-305 was added. The addition of surfactants also reduced the formation of the beads during the preparation of nanofibers. The decrease of diameters and bead formation is attributed to the reduced surface tension of the polymer solution when the surfactant was added [50].

### 3.3. FTIR Analysis

**Figure 5:**
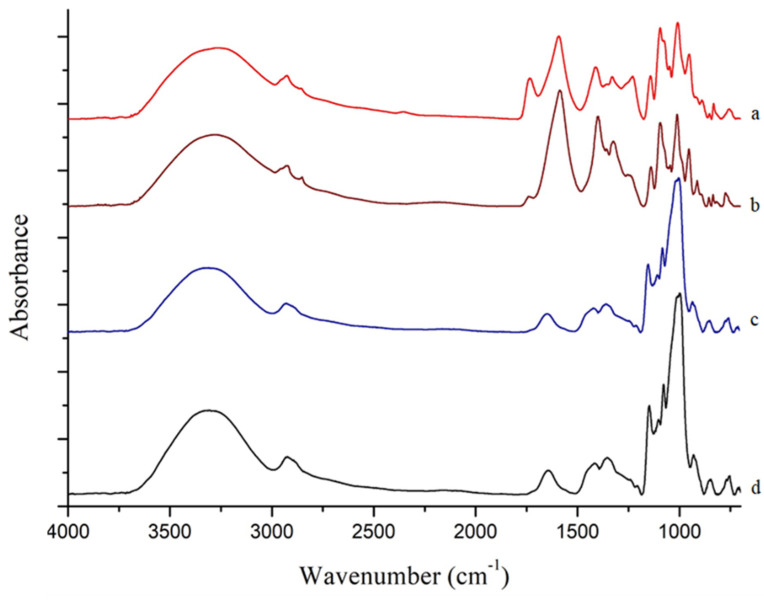
FTIR spectra of JFP and JFP/pullulan nanofibers: (**a**) JFP; (**b**) EDTA extracted JFP; (**c**) pullulan nanofibers; (**d**) JFP/pullulan nanofibers (1J12P1T305).

The functional group analyses of the JFP samples are given in Figure 5a,b. The carbohydrate characteristic was observed between 950–1200 cm**^−^**^1^, which was used to determine the main chemical component. The characteristic peaks observed at 2936 cm**^−^**^1^ correspond to the CH bond of methylene (CH_2_) and the peak at 3443 cm**^−^**^1^ to the OH bond. However, the carboxylate (COO-) and methyl esterified carboxyl (COOMe) groups were noticed at 1615 and 1744 cm**^−^**^1^, respectively. The degree of methylation of polysaccharides can be calculated from the characteristic peak areas of carboxylic acids and methyl esterified carboxyl groups [37]. The degree of methylation of JFP polysaccharide was around 39%, and as the value was less than 50%, the presence of the pectin molecule is low methyl pectin. The JFP obtained either with or without EDTA were low methyl pectin, while the JFP extracted with EDTA had a lower degree of methylation than that extracted without EDTA, attributed to the chelating behavior of EDTA that destroy the bridges between JFP molecules and metal ions during the extraction process and consequentially allowing methylesterase to perform de-esterification [33].

FTIR analysis of pullulan (Figure 5c) showed that the peaks at 754 and 930 cm**^−^**^1^ were attributed to α-(1,4) and α-(1,6) groups. The peaks found at 851, 1658, and 3330 cm**^−^**^1^ represented the structure of α-d-glucopyranose units, the –OCO– group, and the -OH groups. The data was in agreement with previous studies of pullulan [32,36]. The nanofibers prepared with JFP/pullulan showed characteristic peaks of α-(1,4), α-(1,6), and α-d-glucopyranose units at 754, 930, and 851 cm**^−^**^1^ respectively. The FTIR spectrum of JFP/pullulan nanofibers was shown in Figure 5d. The addition of JFP increased the peak area of –OCO– at 1640 cm**^−^**^1^ and confirmed the existence of JFP in the prepared nanofibers.

### 3.4. TGA Analysis

**Figure 6:**
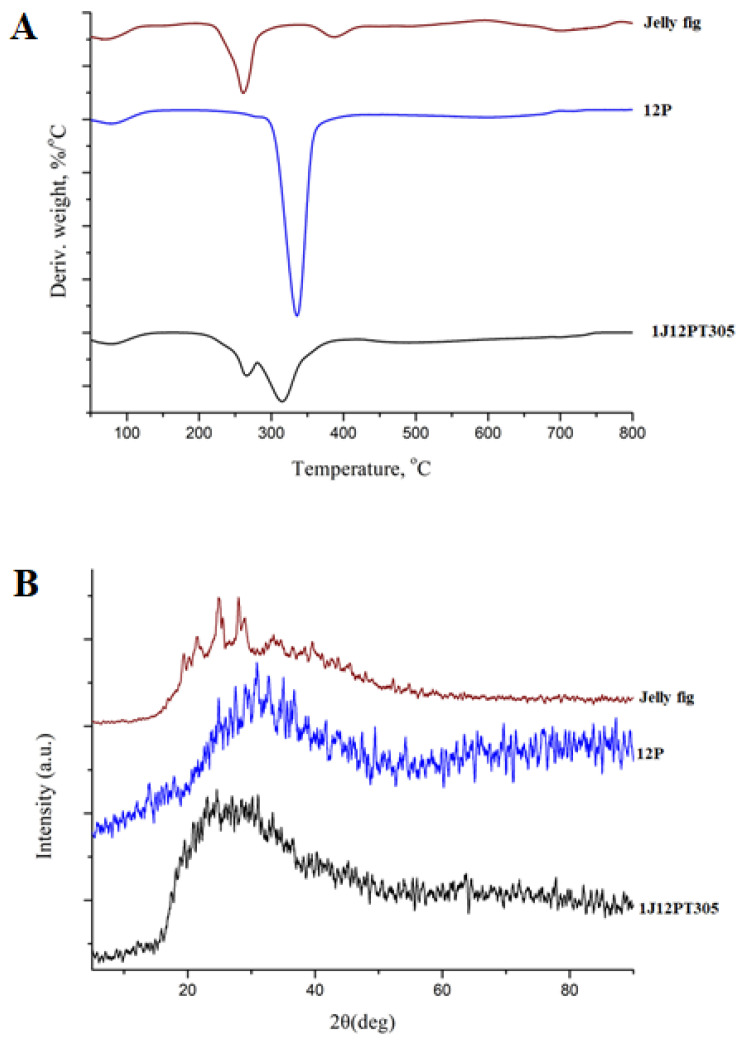
(**A**) Thermogravimetric curves of JFP powder, pullulan and JFP/pullulan nanofibers; (**B**) XRD patterns of JFP, pullulan and JFP/pullulan nanofibers.

The TGA spectra showed the first degradation curve at 100 °C for water loss in JFP and pullulan (Figure 6A). Then, the mass loss of the second stage was determined at 253 °C for JFP and 326 °C of pullulan. However, the JFP/pullulan nanofibers showed three-stage degradation (Figure 6A). The peak noticed at 100 °C corresponded to mass loss of water. The peak observed at 256 °C was deemed the thermal cracking temperature of JFP and the peak at 305 °C as the thermal cracking temperature of pullulan [45]. The TGA results confirmed that the combination of JFP/pullulan nanofiber was successful. Therefore, the addition of JFP to pullulan reduced the thermal cracking temperature in the resulting fiber. The phenomena could have resulted from the decarboxylation or decarbonization of JFP during thermal cracking [51,52].

### 3.5. X-ray Diffraction Analysis

The X-ray diffraction pattern of JFP powder consists of two peaks at 25.1° and 27.9° representing the amorphous and crystalline areas of JFP (Figure 6B). The pullulan was found as a completely amorphous polymer through the X-ray diffraction analysis. The JFP/pullulan nanofiber showed an increased crystalline strength attributed to the interaction between JFP and pullulan molecules through the formation of hydrogen bonds. The JFP and pullulan crystal strength were arranged neatly in the XRD pattern indicated that pullulan was not affected by the addition of JFP [8,53]. FTIR spectroscopy is suitable to illustrate the formation of hydrogen bonding between molecules. Naturally, the hydrogen bond formation would bring a red or blue shift in the neighboring groups of the hydrogen bonds [54]. However, the difference in crystallinity noticed in XRD is the additional probability of hydrogen bond formation for the semi-crystalline polymers [54]. The hydrogen bond formation occurred in the inter JF and pullulan region with increased hard segments. The hydrogen bond formation mainly occurs within the hard segments that stimulate the crystallinity of the polymers [55]. Therefore, the distinct crystallinity was observed in XRD as the reflection of increased hard segments. Similarly, hydrogen bond intensity (HBI) was also used to interpret the qualitative fluctuations in crystallinity [56]. The electrospinning process obstructed the formation of crystals and produced an amorphous crystal structure. The pullulan solution was able to dissolve less water-soluble drugs due to the amorphous nature [57]. Therefore, the nanofibers made by JFP/pullulan can be used as a drug carrier due to the ability of pullulan to generate intermolecular bonds with other materials.

### 3.6. Tensile Strength

**Figure 7:**
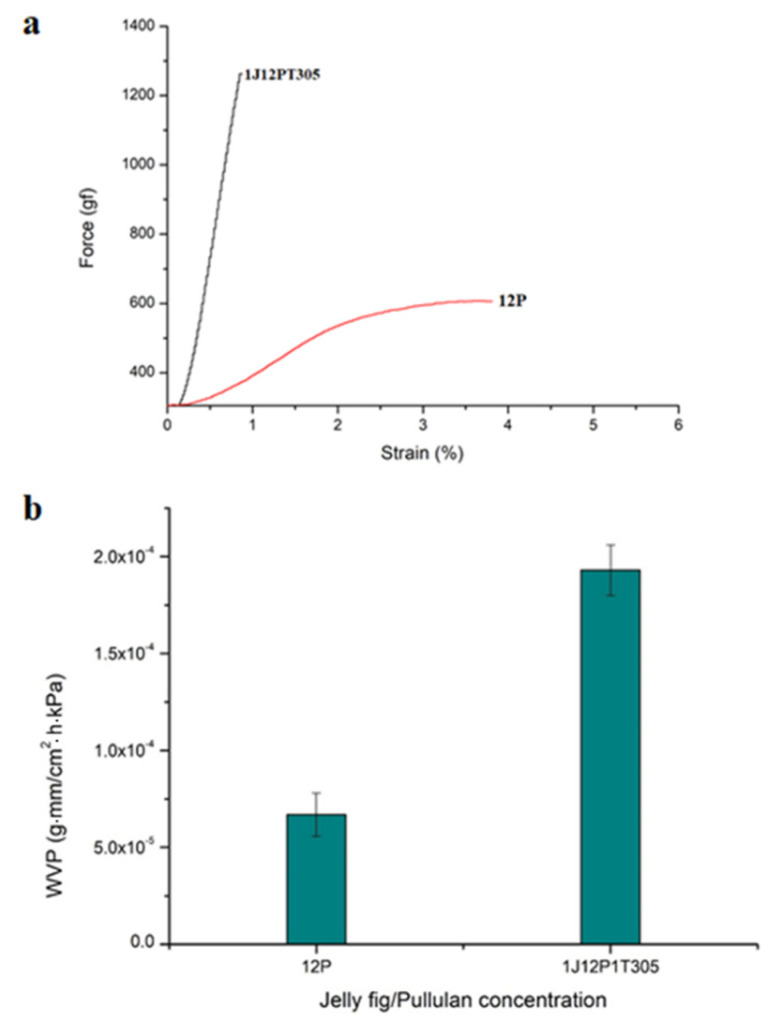
(**a**) The tensile strength of pullulan and JFP/pullulan nanofibers; (**b**) water vapor permeability coefficients of pullulan and JFP/pullulan nanofibers at 25 °C with 75% relative humidity (RH).

The mechanical properties of the JFP/pullulan nanofiber were studied as depicted in Figure 7A. The tensile strength of the pullulan nanofiber was 0.031 ± 0.003 MPa. Pullulan nanofiber possessed good elasticity confirmed by strain behavior of about 3.8%. The presence of α-(1,4) and α-(1,6) unique functional groups in pullulan might improve the elasticity of nanofibers. The addition of JFP to pullulan considerably increased the tensile strength of electrospun nanofibers to 0.051 ± 0.007 MPa. However, the elasticity was reduced accordingly. The results suggested that the JFP can form intermolecular hydrogen bonding with pullulan and improve the tensile strength. The improvement of tensile strength will help JFP/pullulan nanofiber retain good mechanical properties during storage [58].

### 3.7. Water Vapor Permeability (WVP) Measurement

The WVP of pullulan and JFP/pullulan nanofibers were displayed in Figure 7B. The WVP of JFP/pullulan nanofibers was significantly higher than that of pullulan nanofibers. The pullulan nanofibers exhibited a lower WVP value of (6.69 × 10**^−^**^5^, g·mm/Pa·h·cm^2^) than JFP/pullulan nanofibers (1.92 × 10**^−^**^4^, g·mm/Pa·h·cm^2^), which is probably due to the difference in the molecular structure of JFP and pullulan. JFP showed more affinity towards water molecules and increased the water vapor permeability due to the presence of -COOH and –OH groups [59]. Therefore, JFP/pullulan nanofibers exhibited a stronger affinity to water molecules than pullulan nanofibers. Pullulan films also showed low water permeability due to their non-hygroscopic nature [53]. The results revealed that the addition of JFP with pullulan improved the permeability of the nanofiber substantially.

### 3.8. Water Solubility and Dissolution Study of Model Drug-Loaded Nanofibers

**Figure 8:**
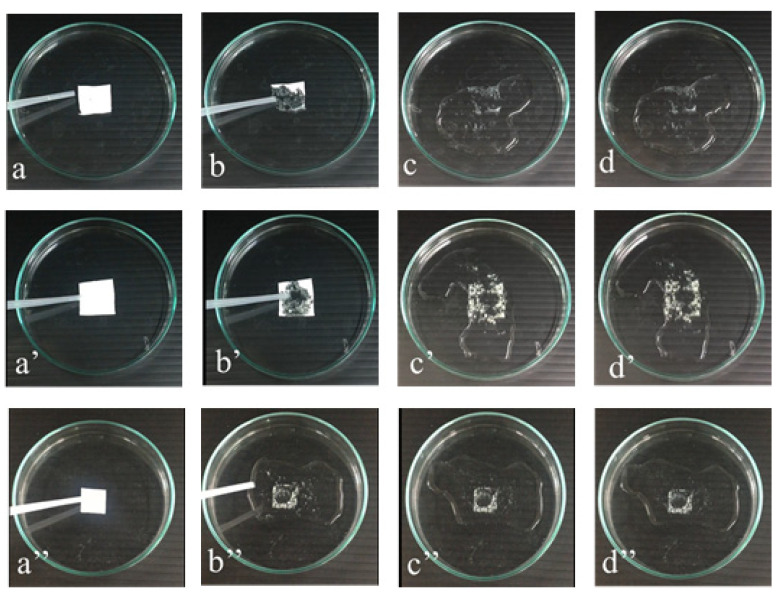
The digital photographs of solubility behavior of pullulan nanofibers (12P), JFP/pullulan nanofibers (1J12P1T305), dexamethasone, and ampicillin loaded JFP/pullulan nanofibers. The photographs were taken before contact with water (**a**,**a’**,**a’’**), and after contact with water for 5 s (**b**,**b’**,**b’’**), 30 s (**c**,**c’**,**c’’**), and 60 s (**d**,**d’**,**d’’**).

Antibiotic and anti-inflammatory drugs are commonly used in wound dressing, but the dissolution of poorly water-soluble drugs is a persisting challenge [57]. Several techniques, such as liposome [60], emulsion, and nanofibers have been continuously developed to overcome this issue. Drug-loaded electrospun nanofibers would be useful for wound dressings to treat infected wounds [61]. The solubility or dissolution property of the prepared nanofibers was investigated using a dissolution test (Figure 8). The solubility and rapid release of the encapsulated model drugs (ampicillin and dexamethasone) were recorded to ensure their solubility, bioavailability, and applicability in an FDDDS (Supplementary Videos S1–S3). The digital photos were taken at predetermined time interval of before contact with water (a-a″), 5 s (b-b″), 30 s (c-c″) and 60 s (d-d″) after contacting with water. The results showed that both pullulan and JFP/pullulan nanofibers were completely dissolved in water within 60 s (1 min), mainly due to the hydrophilic properties of JFP and pullulan, the high surface area to volume ratio, and the nano-scale porosity of electrospun nanofibers [8]. The electrospinning technique played a vital role in converting small crystalline molecules into amorphous molecules during the evaporation of the solvent. Also, the high surface area of the carrier enhanced the drug release considerably by improving the affinity with water molecules and eliminating the amorphous physical nature of the drug [11]. The ampicillin and dexamethasone-loaded JFP/pullulan nanofibers were also dissolved quickly when in contact with water molecules. Therefore, the pullulan nanofibers can be used as a natural fast-dissolving drug carrier.

### 3.9. In Vitro Release Study of Drugs from Prepared Nanofibers

**Figure 9:**
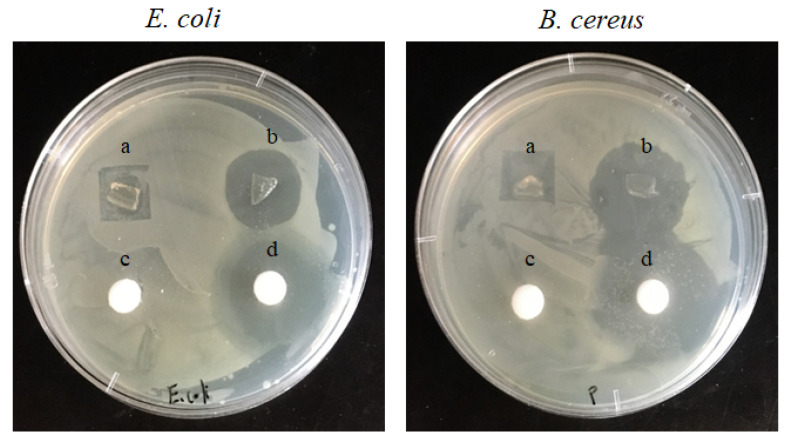
Antibacterial activity of prepared nanofibers showing the zone of inhibition against *E. coli* and *B. cereus* after 8 h of incubation at 37 °C: (**a**) 1J12P1T305; (**b**) dexamethasone and ampicillin loaded JFP/pullulan nanofibers; (**c**) cotton; and (**d**) ampicillin.

The applicability of JFP/pullulan nanofibers in FDDDSs was also assessed by in vitro release of encapsulated model drugs dexamethasone and ampicillin. The antibacterial activity of the drug-loaded nanofibers was tested against both Gram-negative (*E. coli*) and Gram-positive (*B. cereus*) bacteria, and the results are displayed in Figure 9. The drug-loaded JFP/pullulan nanofibers showed a strong antibacterial activity by exhibiting a clear, visible zone of inhibition within 8 h against *E. coli* (left) and *B. cereus* (right) compared to blank JFP/pullulan nanofibers. This study concludes that the electrospinning process did not degrade the ampicillin molecules and improved their water solubility.

**Figure 10:**
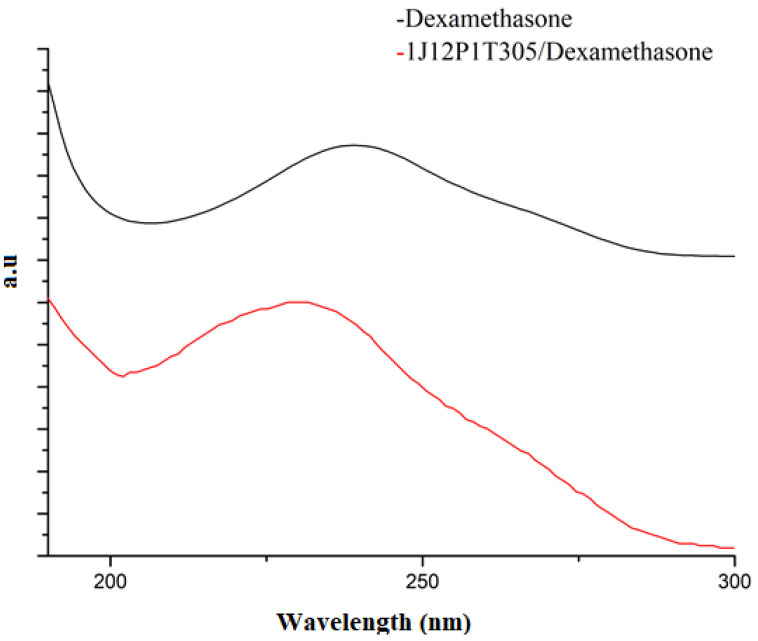
UV/Vis spectra of pure dexamethasone in ethanol (**black**) and dexamethasone loaded JFP/pullulan nanofibers (1J12P1T305) showing the existence (**red**) of dexamethasone in water.

Dexamethasone is well known as an anti-inflammatory drug with anti-allergic capability [62,63]. The low water solubility of dexamethasone made us encapsulate in nanofibers and study its solubility and bioavailability. The nanofiber-based delivery of dexamethasone can be easy and feasible [64]. The electrospinning process ensured the uniform distribution of dexamethasone in nanofibers. The existence of dexamethasone in the JFP/pullulan nanofibers was studied using a UV/Visible spectrometer by dissolving the nanofiber membrane in water. The existence of the dexamethasone was confirmed by a maximum absorption at 240 nm (Figure 10). The pure dexamethasone was subsequently dissolved in ethanol and recorded the absorption maxima at 240 nm for the correlation. The amorphous polymer nature of JFP/pullulan transformed dexamethasone molecules to dissolve quickly in the water. The results confirmed that JFP/pullulan nanofibers would be a promising carrier for the encapsulation of hydrophobic drugs or bioactives to be used in FDDDS.

## 4. Conclusions

Novel fast-dissolving nanofibers were fabricated from the blends of JFP and pullulan by electrospinning. The jelly fig polysaccharides were extracted from achenes of *Ficus pumila var. awkeotsang* using EDTA. Then, jelly fig and pullulan were used to prepare the fast-dissolving nanofibers by electrospinning and characterized thoroughly. The addition of jelly fig to pullulan improved the conductivity of the solution due to the polyelectrolyte property of jelly Figure SEM results confirmed that 1% (*w/w*) jelly fig 12% (*w/w*) pullulan solution along with surfactant Triton-X 305 produced continuous, uniform and bead-free nanofibers with an average fiber diameter of 144 ± 19 nm. FTIR and TGA analysis proved the successful blending of jelly fig and pullulan. The XRD pattern revealed that the formation of intermolecular hydrogen bonds between the jelly fig and pullulan molecule and increased crystallinity. The improved water vapor permeability of jelly fig/pullulan nanofiber was noticed due to the presence of carboxyl and hydroxyl groups in jelly fig moiety. The dissolution test substantiated that jelly fig/pullulan nanofibers can be rapidly soluble in water (60 s). The antibacterial activity and existence of ampicillin/dexamethasone in jelly fig/pullulan nanofibers were also confirmed through the dissolution test. Moreover, the encapsulated hydrophobic drugs can also be quickly dispersed into the surroundings. The results showed that the JFP/pullulan nanofibers can dissolve rapidly in a water medium and could be useful in oral drug delivery. Overall, the present investigation concluded that JFP/pullulan nanofibers could be an alternative material in FDDDSs.

## Data Availability

The data presented in this study are available on request from the corresponding author.

## Supplementary Materials

The following are available online at https://www.mdpi.com/2073-4360/13/2/241/s1, Table S1: Electrospinning formulation, Video S1: Ampicillin and dexamethasone loaded JF/Pullulan nanofiber; Video S2: JF/Pullulan nanofiber; Video S3: Pullulan nanofiber.
