# Supplementary material for: Fast Dissolving Electrospun Nanofibers Fabricated from Jelly Fig Polysaccharide/Pullulan for Drug Delivery Applications
Source: Polymers (Basel). 2021 Jan 12;13(2):241. doi: 10.3390/polym13020241 (PMC7830562; doi:10.3390/polym13020241)
Supplement: Supplementary file 1 [file polymers-13-00241-s001.zip › polymers-1060630 R1 sup.docx]

**Fast dissolving electrospun nanofibers fabricated from jelly fig/pullulan for drug delivery applications**

Thangavel Ponrasu^1^, Bei-Hsin Chen^1^, Jia-Jiuan Wu^2^, and Yu-Shen Cheng^1,*^

^1^ Department of Chemical and Materials Engineering, National Yunlin University of Science and Technology, Douliu, Yunlin 64002, Taiwan

^2^ Department of Nutrition, China Medical University, No. 91, Hsueh-Shih Road, Taichung 404, Taiwan.

**^*^**Corresponding author:

Yu-Shen Cheng, Email: yscheng@yuntech.edu.tw

Phone: (+886) 5534-2601 ext. 4627

Fax: (+886) 5531-2071

**Supplementary information**

**Table S1.** Electrospinning formulation

| Sample code^‡^ | Solution properties  % (w/w) | | | | Solution properties | | |
| --- | --- | --- | --- | --- | --- | --- | --- |
|  | Jelly fig | Pullulan | Triton X-100 | Triton X- 305 | Conductivity  (µS/cm) | Viscosity at 1s^-1^  (Pa^.^s) | Viscosity at 100s^-1^ (Pa^.^s) |
| 10P | - | 10 | - | - | 8.8 ± 0.15 | 0.212 ± 0.02 | 0.142 ± 0.033 |
| 12P | - | 12 | - | - | 27.5 ± 0.1 | 0.391 ± 0.03 | 0.257 ± 0.018 |
| 1J10P | - | 10 | - | - | 302.8 ± 9 | 0.626 ± 0.095 | 0.368 ± 0.065 |
| 1J12P | 1 | 12 | - | - | 407.3 ± 2.2 | 1.162 ± 0.095 | 0.625 ± 0.065 |
| 1J10P1T100 | 1 | 10 | + | - | 705.2 ± 51.4 | 0.467 ± 0.064 | 0.29 ± 0.04 |
| 1J10P1T305 | 1 | 10 | + | - | 946.3 ± 14.4 | 0.475 ± 0.046 | 0.325 ± 0.023 |
| 1J12P1T100 | 1 | 12 | - | + | 751.2 ± 28.1 | 0.859 ± 0.04 | 0.465 ± 0.029 |
| 1J12P1T305 | 1 | 12 | - | + | 1085.7 ± 5.5 | 0.897 ± 0.04 | 0.516 ± 0.04 |

^‡^Sample code: P: Pullulan; J: Jelly fig; T100: Triton X-100; T135: Triton X-135

**Video files**
